# Interventions for healthcare professionals caring for COVID-19 patients (beyond vaccines): A systematic review

**DOI:** 10.1017/ice.2020.323

**Published:** 2020-07-03

**Authors:** Barbara Russo, Michele Iudici

**Affiliations:** 1 Department of Pathology and Immunology, University of Geneva, Switzerland; 2 Geneva University Hospitals, Internal Medicine Specialties, Rheumatology Unit, Geneva, Switzerland

*To the Editor—*As of June 20, 2020, >8 million individual cases of novel coronavirus disease 2019 (COVID-19) infection have been reported worldwide, with ~454,000 reported deaths. This disease is caused by the severe acute respiratory syndrome coronavirus 2 (SARS-CoV-2), similar to severe acute respiratory syndrome coronavirus (SARS-CoV) and Middle East respiratory syndrome coronavirus (MERS-CoV). Currently, no vaccine or proven effective prophylactic treatment is available.^1^

Despite the measures to prevent contamination, many cases of infections and deaths have been observed among healthcare professionals (HCPs)^2^. Furthermore, the high number of severe patients to manage, the increased workload, and the shortage or inadequacy of personal protective equipment (PPE), are sources of physical and psychological stress for HCPs.

All of these factors may contribute to the shortage of personnel working in healthcare facilities, with potential detrimental impact on the health system. Therefore, it is crucial to identify interventions to support and protect HCPs. A summary of preventative or therapeutic interventions specifically designed for HCPs facing the COVID-19 pandemic, could help to drive future research in the field.

## Methods

We followed the PRISMA reporting guidelines for systematic reviews and meta-analysis.^3^

On June 18, 2020, the terms “COVID” OR “coronavirus” were used to search the World Health Organization International Clinical Trials Registry Platform (ICTRP)^4^ for all interventional studies registered since December 31, 2019, when the first case of COVID-19 was declared in China.

Two authors independently screened titles and the full content of the registered studies in duplicate. We included all studies testing interventions for HCPs exposed or at risk to be exposed to COVID-19 patients. Studies on COVID-19 recruiting, as well as other categories of workers or healthy people, were included only if the enrollment of HCPs was clearly indicated in eligibility criteria. One reviewer extracted information from eligible studies, and a second reviewer verified the extraction. Disagreements were discussed to reach consensus.

## Results

Among the 2,161 studies retrieved, 56 (2.6%) were designed to test pharmacological (n = 44) or nonpharmacologic (n = 12) treatments in eligible population. Trials were registered in the United States (n = 15), Spain (n = 10), China (n = 9), Canada (n = 5), the United Kingdom (n = 5), Brazil (n = 2), Mexico (n = 2), Pakistan (n = 2), Argentina (n = 1), Colombia (n = 1), France (n = 1), Hungary (n = 1), Iran (n = 1), and The Netherlands (n = 1). These studies were mostly randomized (n = 45; 80.3%), placebo-controlled (n = 32; 57.2%), intended to enroll median 450.5 participants (interquartile range [IQR], 237.5–1,475; range, 40–55,000). Most studies investigated interventions given to prevent COVID-19 infection in healthy HCPs (n = 41; 73.2%). Mental health status (eg, depression, anxiety, mood disorders) (n = 7; 12.5%), the rate of absenteeism (n = 3; 5.4%), and the disease evolution for infected HCPs (n = 3; 5.4%) are the primary end points of the remaining studies (Table 1).

Among the pharmacological interventions, antimalarials drugs (chloroquine [CQ], hydroxychloroquine [HCQ], or mefloquine [MFQ]) used as pre- or postexposure preventative treatment, are the compounds more commonly investigated (n = 26; 46.4%). One study assesses the severity of the COVID-19 disease in symptomatic HCPs receiving HCQ or not receiving HCQ. The proposed dose, the schemes of administration, and the treatment duration are quite heterogenous. For HCQ, dose ranges from 200 to 800 mg daily, and some studies used a loading dose followed by a maintenance regimen. The durations of treatment ranged between 3 days and 6 months (see the supplementary file online). Other pharmacologic interventions under study include human interferon (α-1b and β-1a), antituberculosis Bacille Calmette-Guérin (BCG) vaccine, nitric oxide, lopinavir–ritonavir, emtricitabine–tenofovir, povidone iodine, nitazoxanide, GLS-1200, *Lactobacillus*, and convalescent plasma. All of these interventions are used to prevent or reduce the severity of COVID-19 (see Supplementary Material online).

The nonpharmacological interventions under study are devices for personal protection (surgical masks vs N95 respirators; gastroscope isolation mask), and psychological, educational, or behavioral therapies aiming to decrease the anxiety, depression, or sleep problems of people involved in the care of COVID-19 patients. Finally, traditional Chinese medicine or rehabilitation practices, alone or in combination with other interventions, are under investigation in 2 studies conducted to improve mental health and well-being of nurses and physicians (see Supplementary Material online).

Our review has several limitations. The enormous number of trials registered every day, in different platforms worldwide, could very quickly modify the landscape of ongoing research. Additionally, we could have missed some ongoing studies on nonpharmacologic interventions for which protocols have not been registered.

In conclusion, since the identification of the first case of COVID-19 in China, a growing number of trials is being conducted to test interventions for HCPs working every day in close contact with (potentially) infected patients. Findings from this review reveal that the prophylactic use of antimalarials raises high expectations in the scientific community. Due to the heterogeneity in dose, duration of treatments, methods chosen to enroll participants, and methods chosen to ascertain the infection, interpretation of the future results on antimalarials should be critically evaluated. By contrast, less is being done to test the performance of personal protection equipment, to identify educational strategies to be delivered to HCPs to minimize their risk of infection, or to help them to cope with chronic stress.

**Table 1.** Summary of the Main Characteristics of the Trials Included

Item and Subcategory	Overall Studies (N = 56)	
	No.	%^a^
**Location of sponsor**		
North America	22	39.3
Europe	19	33.9
Asia	11	19.6
South America	4	7.2
**Participants** ^b^		
HCPs (no distinction of role)	49	87.4
Only nurses	2	3.6
Only physicians	2	3.6
Only nurses and physicians	2	3.6
Healthcare students	1	1.8
**Type of intervention**		
**Pharmacologic**	**44**	**78.6**
Antimalarials (HCQ, CQ, or MFQ) (in 3 studies combined with other treatments)^c^	26	46.4
BCG vaccine	5	8.9
Rh-INFα-1b/IFN β-1A	3	5.3
Nitric oxide	2	3.6
Povidone iodine	2	3.6
Lopinavir/ritonavir	1	1.8
Emtricitabine/Tenofovir	1	1.8
Nitazoxanide	1	1.8
GLS-1200	1	1.8
Lactobacillus	1	1.8
Convalescent plasma	1	1.8
**Nonpharmacologic**	**12**	**21.4**
Psychologic or educational or behavioral or rehabilitation	7	12.4
Devices	2	3.6
Traditional Chinese therapy	2	3.6
Other	1	1.8
**Study design**		
Randomized	45	80.3
Nonrandomized	11	19.7
**Type of comparator**		
Placebo	32	57.2
No intervention	12	21.4
Active (nonpharmacologic)	3	5.3
Active (pharmacologic)	1	1.8
Usual care	1	1.8
Single arm	5	8.9
Unclear	2	3.6
**Time to primary outcome, median d and range**	45.5	14–180
**Sample size**		
No. of patients planned to be included or included per study, median and IQR and range	450.5	237.5–1,475; 40–55,000

Note. BCG, Bacille Calmette-Guerin; CQ, chloroquine; HCPs, healthcare professionals; HCQ, hydroxychloroquine; IQR, interquartile range; MFQ, mefloquine; Rh-INF, recombinant human interferon.

a If not specified otherwise.

b 12 studies included also other categories of at risk-people.

c HCQ + vitamins C, D, and zinc in 1 single-arm study; HCQ + bromhexine in 1 arm of a 2-arm study; HCQ + emtricitabine/tenofovir disoproxil in 1 arm of a 4-arm study.

## Supplementary material

For supplementary material accompanying this paper visit http://dx.doi.org/10.1017/ice.2020.323. click here to view supplementary material
