# Supplementary material for: Interventions for healthcare professionals caring for COVID-19 patients (beyond vaccines): A systematic review
Source: Infect Control Hosp Epidemiol. 2020 Jul 3:1–2. doi: 10.1017/ice.2020.323 (PMC7369340; doi:10.1017/ice.2020.323)
Supplement: Supplementary file 1 [file S0899823X20003232sup001.docx]

**Online supplementary file.**

**Table 1.** Summary of the main characteristics of the trials investigating pharmacologic interventions.

| **Trial ID** | **Population** | **Intervention(s)** | **Primary outcome(s)** | **Time**  **to PO** | **Size** | **Randomized** | **Location of the sponsor** |
| --- | --- | --- | --- | --- | --- | --- | --- |
| **Antimalarials (HCQ, CQ, MFQ)** | | | | | | | |
| NCT04318015  PHYDRA | HCPs exposed to COVID-19 patients | Arm 1: HCQ 200 mg/day for 60 days  Arm 2: Placebo | N of symptomatic  COVID-19 infections | 60 days | 400 | Yes | Mexico |
| NCT04303507  COPCOV | HCPs at risk to be exposed or exposed to COVID-19 patients* | Arm 1: HCQ or CQ loading dose 10 mg/kg base followed by 155 mg base^†^day for 3 months  Arm 2: Placebo | N of symptomatic COVID-19 infections;  Symptoms severity of COVID-19 | 100 days | 40000 | Yes | UK |
| NCT04328961 | HCPs exposed to COVID-19 patients * | Arm 1: HCQ 400 mg/day for 3 days, then 200 mg/day for additional 11 days  Arm 2: Placebo | PCR confirmed SARS-CoV-2 infection | 14, 28 days | 2000 | Yes | USA |
| NCT04330144  HOPE | HCPs exposed to COVID-19 patients* | Arm 1: HCQ 800 mg/day for 1 day, then 400 mg/day for additional 4 days  Arm 2: No intervention | PCR confirmed SARS-CoV-2 infection | 14 days | 2486 | Yes | China |
| NCT04343677 | HCPs at risk to be exposed or exposed to COVID patients* | Arm 1: HCQ 200 mg/day  (low dose) for not exposed  Arm 2: 400 mg/day (high dose) for not exposed  Arm 3: 400 mg/day for the exposed  Treatment duration: 7 days (specified for post-exposure arm)  Arm 4: Placebo | Incidence of  COVID-19 infection | 60 days | 1450 | Yes | USA |
| NCT04335084  HELPCOVID-19 | HCPs exposed to COVID-19 patients | Arm 1: HCQ, vitamines C, D, Zinc | Prevention of COVID-19 measured by negative testing with PCR  Safety as determined by blood pressure readings or by presence of side effects | 24 weeks | 600 | No | USA |
| NCT04308668  COVID-19 PEP | HCPs exposed to COVID-19 patients or symptomatic for COVID-19 disease* | Arm 1: HCQ 800 mg orally once, followed in 6 to 8 hours by 600 mg, then 600 mg/day for 4 days  Arm 2: Placebo | Incidence of COVID19 and self-report disease severity status among those who are asymptomatic at trial entry | 14 days | 3000 | Yes | USA |
| NCT04304053  HCQ4COV19 | HCPs exposed to COVID-19 patients* | Arm 1: HCQ 800 mg/day for 1 day, then 400 mg/day for additional 3 days  Arm 2: No treatment | Incidence of COVID-19 infection | 14 days | 3040 | Yes | Spain |
| NCT04331834  PrEP-COVID | HCPs at risk to be exposed to COVID-19 patients | Arm 1: HCQ 200 mg twice daily for 4 days, then 400 mg weekly for 6 months  Arm 2: Placebo | Incidence of COVID-19 infection | 6 months | 440 | Yes | Spain |
| NCT04333732  CROWN CORONA | HCPs at risk to be exposed to COVID-19 patients | Arm 1: 300 mg base/weekly  Arm 2: 300 mg base twice weekly  Arm 3 :150 mg base daily  Placebo | Symptomatic COVID-19  Peak severity of COVID-19 over the study period | 3 months | 55000 | Yes | USA |
| NCT04340349  HCQINRLGII | HCPs exposed to COVID-19 patients | Arm 1: HCQ 200 mg/day + Bromhexine every 8 hrs for 2 months  Arm 2: Bromhexine every 8 hrs for 2 months | PCR confirmed SARS-CoV-2 infection | 30 and 60 days | 100 | Yes | Mexico |
| NCT04333225 | HCPs exposed to COVID-19 patients | Arm 1: HCQ 400 mg twice a day on day 1 followed by two 200 mg tablets once a week for a total of 7 weeks  Arm 2: No intervention | PCR confirmed SARS-CoV-2 infection | 7 weeks | 360 | No | USA |
| NCT04334148  HERO-HCQ | HCPs exposed to COVID-19 patients | Arm 1: HCQ 600mg twice a day on day 1 followed by 400 mg on days 2-30  Arm 2: Placebo | Symptomatic SARS-CoV-2 infection | 30 days | 15000 | Yes | USA |
| EUCTR2020-001421-31-ES | HCPs at risk to be exposed to COVID-19 patients | Arm 1: HCQ°  Arm 2: HCQ°  Arm 3: HCQ° | Symptomatic SARS-CoV-2 infection | ? | 1513 | Yes | Spain |
| EUCTR2020-001194-69-ES  MEFLOCOVID-19 | HCPs exposed to COVID-19 patients* | Arm 1: MFQ 250 mg/day for 30 days  Arm 2: Placebo | SARS-CoV-2 infection | 30 days | 200 | Yes | Spain |
| NCT04334928  EPICOS | HCPs at risk to be exposed to COVID-19 patients | Arm 1: Emtricitabine/ Tenofovir disoproxil, 200/245 mg/day + placebo  Arm2: HCQ 200 mg/day + placebo  Arm 3: Tenofovir disoproxil, 200/245 mg/day + HCQ 200 mg/day  Arm4: double placebo | Symptomatic SARS-CoV-2 infection | 12 weeks | 4000 | Yes | Spain |
| NCT04363450  HCQPreP | HCPs exposed to COVID-19 patients | Arm 1: HCQ 400mg (2 cps) twice 12 hours apart followed by 200mg twice weekly for 12 weeks  Arm 2: Placebo | PCR confirmed SARS-CoV-2 infection in symptomatic patients | 12 weeks | 1700 | Yes | USA |
| NCT04371523  PROVIDE | HCPs exposed to COVID-19 patients | Arm 1: HCQ 400 mg twice a day on day 1, then 400 mg weekly for two months.  Arm 2: Placebo | PCR (or antigen test) confirmed SARS-CoV-2 infection | 8 weeks | 1100 | Yes | Canada |
| NCT04359537  CHEER | HCPs exposed to COVID-19 patients | Arm 1: HCQ 400mg twice a day on day 1 followed by 400 mg once a week for a total of 12 weeks  Arm 2: HCQ 400 mg on day 1 followed by 400mg once every 3 weeks for a total of 12 weeks  Arm 3: HCQ 200 mg on day 1 followed by 200 mg once every 3 weeks for a total of 12 weeks  Arm 4: Placebo | COVID-19-free survival | 12 weeks | 200 | Yes | Pakistan |
| NCT04370015 | Physicians and nurses exposed to COVID-19 patients | Arm 1: HCQ 400 mg twice a day on day 1 followed by 400mg once a week for 11 weeks.  Arm 2: Placebo | PCR confirmed SARS-CoV-2 infection  Safety | 12 weeks | 374 | Yes | Pakistan |
| EUDRACT 2020-001704-42  SANsinCOVID | HCPs exposed to COVID-19 patients | Arm 1: HCQ for 2 months  Arm 2: Placebo | Symptomatic or asymptomatic SARS-CoV-2 infection  Severity of infection | 2 months | 450 | Yes | Spain |
| EUDRACT2020-001536-98  PREPSARS | HCPs exposed to COVID-19 patients | Arm 1: HCQ prophylaxis  Arm 2: No intervention | PCR confirmed SARS-CoV-2 symptomatic infection | 30 days | 300 | No | Spain |
| NCT04384458 | HCPs exposed to COVID-19 patients | Arm 1: HCQ 400 mg twice a day on day 1, one 400 mg tablet on day 2, 3, 4, and 5, followed by one 400 mg tablets every 5 days until day 50^th^ associated with 66 mg of zinc sulfate  Arm 2: No intervention | PCR (or antigen test) confirmed SARS-CoV-2 infection | 50 days | 400 | Yes | Brazil |
| NCT04372017 | HCPs exposed to COVID-19 patients* | Arm 1: HCQ 800mg on day 1 followed by 400mg on days 2-5  Arm 2: Placebo | PCR confirmed SARS-CoV-2 infection | ? | ? | Yes | USA |
| NCT04421664  COVID-19 PEP Canada | Symptomatic HCPs exposed to COVID-19 patients | Arm 1: HCQ 800 mg orally once, followed in 6 to 8 hours by 600 mg, then 600mg once a day for 4 consecutive days  Arm 2: Placebo | Ordinal Scale of COVID19 Disease Severity | 14 days | 1500 | Yes | Canada |
| EUDRACT 2020-001440-26  PrevenCOVID-19 | HCPs (expected to be) exposed to COVID-19 patients | Arm 1: HCQ  Arm 2: Placebo | PCR confirmed SARS-CoV-2 infection | 60 days | 184 | Yes | Spain |
| **Other agents** |  |  |  |  |  |  |  |
| NCT04328441 | HCPs (expected to be) exposed to COVID-19 patients | Arm 1: 0.1ml BCG vaccine (0.075 mg of attenuated Mycobacterium bovis)  Arm 2: Placebo | N of unplanned absenteeism days for any reason | 6 months | 1500 | Yes | The Netherlands |
| EUDRACT 2020-001783-28 | HCPs exposed to COVID-19 patients | Arm 1: BCG vaccine (Danish strain 1331, SSI, Denmark)  Arm 2: Placebo | N of days of unplanned absenteeism because of documented COVID-19 infection | 6 months | 1000 | Yes | Hungary |
| NCT04384549 | HCPs exposed to COVID-19 patients | Arm 1: One intradermal injection of 0.1 ml of BCG vaccine (AJ Vaccine)  Arm 2: Placebo | Confirmed COVID-19 by either nasopharyngeal tests and/or by thoracic CT and/or seroconversion | 6 months | 1120 | Yes | France |
| **IRCT20200411047019N1** | HCPs (expected to be) exposed to COVID-19 patients | Arm 1: 0.10 ml of BCG vaccine  Arm 2: Placebo | Confirmed COVID-19  Hospitalization  Death | 12 months | 500 | Yes | Iran |
| NCT04362124 | HCPs exposed to COVID-19 patients | Arm 1: 0.1 ml of between 1 x 105 to 33 x 105 CFU of BCG.  Arm 2: Placebo | New onset of probable or confirmed COVID-19 disease | 360 | 1000 | Yes | Colombia |
| NCT04405271  CoviPrep | HCPs (expected to be) exposed to COVID-19 patients | Arm 1: Emtricitabine/Tenofovir alafenamide in 200 mg/25 mg tablets. One tablet per day for 12 weeks  Arm 2: Placebo | PCR confirmed SARS-CoV-2 symptomatic infection | 12 weeks | 1378 | Yes | Argentina |
| NCT04321174  CORIPREV-LR | HCPs exposed to COVID-19 patients | Arm 1: Lopinavir/Ritonavir  400/100 mg twice daily for 14 days  Arm 2: No intervention | PCR confirmed SARS-CoV-2 infection | 14 days | 1220 | Yes | Canada |
| NCT04337918  NOCOVID | HCPs exposed to COVID patients* | Arm 1: NORS (gargle every morning, nasopharyngeal irrigation every evening, and NO nasal spray up to 5 times per day) + SOC for 21 days  Arm 2: SOC | PCR confirmed SARS-CoV-2 infection or COVID-19 symptom (fatigue with either fever >37.2 (oral)and/or a persistent cough) | 21 days | 200 | Yes | Canada |
| NCT04312243  NOpreventCOVID | HCPs exposed to COVID-19 patients | Arm 1: Inhaled NO (160 ppm) before and after the work shift for 14 days  Arm 2: No intervention | PCR confirmed SARS-CoV-2 infection | 14 days | 470 | Yes | USA |
| ChiCTR2000030013 | HCPs exposed to COVID-19 patients | Arm 1: Rh-INFα-1b spray  Arm 2: No intervention | Positive rate of new SARS-CoV-2 detected at PCR  positive serology, incidence of severe/critical pneumonia | 6 months | 450 | No | China |
| NCT04320238 | Physicians and nurses at risk to be exposed or exposed to COVID-19 patients* | Arm 1: Rh-INFα-1b  nasal drops, 2-3 drops for each nostril per time, 4 times per day  (for unexposed)  Arm 2: Rh-INFα-1b +  Thymosin a1, 1 sc injection weekly (for exposed) | New onset COVID-19 disease | 6 weeks | 2944 | No | China |
| NCT04385095 | HCPs exposed to COVID-19 patients* | Arm 1: Interferon Beta-1A via inhalation  Arm 2: Placebo | Clinical improvement | Day 1 to 28 | 400 | Yes | UK |
| NCT04366180 | HCPs exposed to COVID-19 patients | Arm 1: 1 cps of Lactobacillus K8 per day (3x10^9 cfu/day) for 2 months  Arm 2: Placebo | PCR (or antigen test) confirmed SARS-CoV-2 infection | 8 weeks | 314 | Yes | Spain |
| NCT04408183 | HCPs (expected to be) exposed to COVID-19 patients | Arm 1: 1 mL of GLS-1200 per nostril  Arm 2: Placebo | PCR confirmed SARS-CoV-2 infection  Safety | 4 weeks | 225 | Yes | US |
| NCT04364802  (PIIPPI) | HCPs exposed to COVID-19 patients* | Arm 1:  Povidone-Iodine Nasal Spray and Gargle  Arm 2: No intervention | PCR confirmed SARS-CoV-2 infection | 3 weeks | 250 | No | USA |
| NCT04393792  SINUS WASH | HCPs with as positive COVID-19 test* | Arm 1: Povidone-Iodine 0.23% sinus rinse and mouthwash three times daily (tds) for days 1-3 of study  Arm 2 : Placebo | Change in viral load in the oral and nasopharyngeal cavity | 14 days | 40 | Yes | UK |
| NCT04390503 | HCPs exposed to COVID-19 patients without PPE* | Arm 1: 1 unit (approximately 200 to 250 mL) of convalescent plasma  Arm 2: Albumin | Rate of severe disease | 28 days | 200 | Yes | USA |
| NCT04359680 | HCPs (expected to be) exposed to COVID-19 patients | Arm 1: Nitazoxanide 600 mg twice daily for six weeks  Arm 2: Placebo | The proportion of subjects with symptomatic laboratory-confirmed COVID-19 or viral respiratory illnesseses | 6 weeks | 800 | Yes | USA |

**BCG.** Bacillus Calmette-Guérin ; **CQ.** Chloroquine; **HCPs.** Health care professionals; **HCQ.** Hydroxychloroquine; **MFQ.** Mefloquine; **N.** Number; **NA.** Not applicable; **NO.** Nitric oxide; **NORS.** Nitric Oxide Releasing Solution; **PCR.** Polymerase chain reaction; **PO.** Primary outcome; **ppm.** Parts per million; **Rh-INFα-1b.** Recombinant human interferon α-1b; **SARS-CoV-2**. Severe acute respiratory syndrome coronavirus 2; **sc.** subcutaneous; **SOC.** Standard of care.

* The trial enrolls also at-risk non-HCPs individuals.

° Explication not provided by the authors.

^†^ 250 mg of Chloroquine phosphate is equivalent to 150 mg base^,^ 200 mg of hydroxychloroquine is equivalent to 155 mg base

**Table 2**. Summary of the main characteristics of the trials investigating non-pharmacologic interventions.

| **Trial ID** | **Population** | | | **Intervention(s)** | **Primary outcome(s)** | | **Time**  **to PO** | **Size** | **Randomized** | **Location of the sponsor** |
| --- | --- | --- | --- | --- | --- | --- | --- | --- | --- | --- |
| **Devices** | | | | | | | | | | |
| NCT04296643 | Nurses exposed to COVID-19 patients | | | Arm 1: Surgical mask during non-aerosol generating procedures  Arm 2: N95 respirator during non-aerosol generating procedures | PCR confirmed SARS-CoV-2 infection | | 6 months | 576 | Yes | Canada |
| ChiCTR2000030317 | Physicians at risk to be exposed or exposed to COVID-19 patients | | | Arm 1: Self-made gastroscope mask during gastroscopy  Arm 2: No intervention | SARS-CoV-2 infection among medical staff (secondary outcome) | | ? | 300 | Yes | China |
| **Other interventions** | | | | | | | | | | |
| ChiCTR2000030432 | | Nurses exposed to COVID-19 patients | Arm 1: Rehabilitation and lung eight-segment exercise  Arm 2: No intervention | | | PCL index^++^  PSQI  FSI^++^ | NA | 80 | Yes | China |
| NCT04283838 | | HCPs exposed to COVID-19 patients | Arm 1: Psychological and physical rehabilitation based humanistic care regimen | | | Self-rating depression scale | 30 days | - | No | China |
| ChiCTR2000030093 | | HCPs exposed to COVID-19 patients | Arm 1: Simplified CBT  Arm 2: Supportive psychotherapy | | | State anxiety | NA | 60 | No | China |
| ChiCTR2000029639 | | Physicians and nurses exposed to COVID-19 patients | Arm 1: Psychological intervention | | | Physical examination  GAD-7  PHQ-9  SASRQPSQI  SARS-CoV-2 detected at PCR  Lymphocyte count | NA | 196 | No | China |
| ChiCTR2000030386 | | Physicians and others at risk to be exposed or exposed to COVID-19 patients* | Arm 1: Moxibustion  Arm 2: ? | | | Mood assessment self-assessment scale  Moxa related Questionnaire - Pneumonia related issues | 14 days | 1000 | ? | China |
| - UTN Number U1111-1251-0177 | | HCPs exposed to COVID-19 patients with respiratory symptoms and fever | Arm 1: Immediate removal from workplace for 6 days  Arm 2: Immediate removal from workplace for 14 days | | | Absenteeism | ? | 250 | Yes | Brazil |
| NCT04410016  COVID-WELL | | HCPs (expected to be) exposed to COVID-19 patients | Arm 1: Staff wellbeing centre | | | Warwick-Edinburgh Mental Wellbeing Scale | Baseline | 45 | No | UK |
| NCT04429828  PoWerS | | Healthcare students | Arm 1: COVID-19 educational learning package around psychological wellbeing | | | Qualitative Interviews with Healthcare Students  Warwick-Edinburgh Mental Wellbeing Scale | 6 weeks | 45 | No | UK |
| NCT04370938 | | HCPs (expected to be) exposed to COVID-19 patients | Arm 1: Coping stress strategy video  Arm 2: No intervention | | | Feasibility of undertaking task | 6 months | 250 | Yes | USA |
| NCT04393818  PsyCovid_App | | HCPs exposed to COVID-19 patients | Arm 1: An app will be used to deliver psychoeducational materials  Arm 2: Sham app | | | Depression, anxiety and stress | 2 weeks | 560 | Yes | Spain |

**CBT.** Cognitive-behavioral therapy; **GAD-7**. Generalized Anxiety Disorder 7; **HCPs**. Healthcare professionals; **PHQ-9**. Patient Health Questionnaire-9 (for depression); **PO.** Primary outcome; **PSQI**. Pittsburgh Sleep Quality Index; **SARS-CoV-2.** Severe acute respiratory syndrome coronavirus 2; **SASRQ.** Stanford Acute Stress Reaction Questionnaire

++ Explication not provided by the authors.
